# Elevated CO_2_ shifts the functional structure and metabolic potentials of soil microbial communities in a C_4_ agroecosystem

**DOI:** 10.1038/srep09316

**Published:** 2015-03-20

**Authors:** Jinbo Xiong, Zhili He, Shengjing Shi, Angela Kent, Ye Deng, Liyou Wu, Joy D. Van Nostrand, Jizhong Zhou

**Affiliations:** 1 Faculty of Marine Sciences, Ningbo University, Ningbo, 315211, China; 2 Institute for Environmental Genomics and Department of Microbiology and Plant Biology, the University of Oklahoma, Norman, OK 73019; 3 Department of Environmental Science, Policy and Management, University of California, Berkeley, CA 94720; 4 Department of Natural Resources and Environmental Sciences, University of Illinois at Urbana-Champaign, Urbana, IL 61820; 5 Key Laboratory of Environmental Biotechnology, Research Center for Eco-Environmental Sciences, CAS, 100085, China; 6 State Key Joint Laboratory of Environment Simulation and Pollution Control, School of Environment, Tsinghua University, Beijing 100084, China; 7 Earth Sciences Division, Lawrence Berkeley National Laboratory, Berkeley, CA 94720

## Abstract

Atmospheric CO_2_ concentration is continuously increasing, and previous studies have shown that elevated CO_2_ (eCO_2_) significantly impacts C_3_ plants and their soil microbial communities. However, little is known about effects of eCO_2_ on the compositional and functional structure, and metabolic potential of soil microbial communities under C_4_ plants. Here we showed that a C_4_ maize agroecosystem exposed to eCO_2_ for eight years shifted the functional and phylogenetic structure of soil microbial communities at both soil depths (0–5 cm and 5–15 cm) using EcoPlate and functional gene array (GeoChip 3.0) analyses. The abundances of key genes involved in carbon (C), nitrogen (N) and phosphorus (P) cycling were significantly stimulated under eCO_2_ at both soil depths, although some differences in carbon utilization patterns were observed between the two soil depths. Consistently, CO_2_ was found to be the dominant factor explaining 11.9% of the structural variation of functional genes, while depth and the interaction of depth and CO_2_ explained 5.2% and 3.8%, respectively. This study implies that eCO_2_ has profound effects on the functional structure and metabolic potential/activity of soil microbial communities associated with C_4_ plants, possibly leading to changes in ecosystem functioning and feedbacks to global change in C_4_ agroecosystems.

Atmospheric carbon dioxide (CO_2_) has been increasing at an accelerated pace since the Industrial Revolution, and is nearly 40% higher than it has been at any other time in the last 20 million years^1^. Such increases in CO_2_ concentration can affect, generally indirectly, soil microbial communities and their functions^2^,^3^,^4^, and subsequently, their mediated carbon (C) and nutrient cycling^5^,^6^,^7^. As soil contains the largest terrestrial C pool, shifts in microbial functional potential/activity may have great consequences in C stabilization and storage in soil, leading to either C sequestration or loss^2^,^8^,^9^. Therefore, understanding soil microbial responses to eCO_2_ is important for better predicting the contribution of terrestrial ecosystems to future climate^8^.

Unlike C_3_ plants, elevated CO_2_ (eCO_2_) should not directly stimulate the net CO_2_ assimilation rate of C_4_ plants, as C_4_ photosynthetic pathway is already CO_2_-saturated under current CO_2_ conditions^10^,^11^,^12^. However, eCO_2_ may indirectly promote C_4_ plant growth by increasing soil moisture^10^. Compared to C_3_ plants, our understanding of CO_2_ effects on C_4_ plants and their associated soil microbial communities is very limited. Although C_4_ plants only contribute ~25–30% of the global terrestrial productivity, many of them are ecologically and economically important (e.g., maize for grain, sugarcane and switchgrass for biofuel), and their cultivation is expected to increase in the future^13^,^14^. Therefore, it is necessary to understand the response of soil microbial communities to eCO_2_ in C_4_ agroecosystems.

The impact of eCO_2_ on the belowground microbial community is expected to be largely indirect, mediated through changes in soil nutrients, e.g., C, nitrogen (N) and soil properties^3^,^15^. As soil physiochemical parameters (e.g., nutrient availability, temperature, soil moisture) vary along the soil depth^16^,^17^,^18^, microbial communities may respond to eCO_2_ differently at different depths. Indeed, previous studies in other ecosystems have shown that eCO_2_ produces different effects on the microbial functional genes between soil depths (0–5 cm and 5–15 cm). For example, eCO_2_ significantly stimulated the abundances of many genes involved in C degradation and N cycling in the soil depth of 0–5 cm, but a majority of these genes remained unchanged in the depth of 5–15 cm^7^, indicating microbial responses to eCO_2_ differ along soil depths. Another study reported that soil organic C and N significantly increased in the soil depth of 5–15 cm, but remained unchanged in the depth of 0–5 cm under eCO_2_ in comparison to ambient CO_2_^19^. However, most studies have examined the impact of eCO_2_ on soil microbial communities only at one depth (e.g., 0–15 cm). To fully understand the impact of eCO_2_ on soil microbial communities and their ecosystem processes, it is necessary to examine the response of soil microbial communities on a finer scale (e.g., different depths).

Maize (*Zea mays* L.) is the third most important food crop globally^20^. To discover the effect of eCO_2_ on the agronomy and productivity of important crops in the Midwestern USA, a free air CO_2_ enrichment experimental site (SoyFACE) was established in 2001 in a corn-soy agroecosystem (http://www.igb.illinois.edu/soyface/). In this study, we examined the response of soil microbial communities to maize fumigated with eCO_2_ in this FACE experiment. We hypothesized that eCO_2_ would alter the functional composition, structure and metabolic potential of soil microbial communities associated with maize cultivation, and that various microbial functional groups (e.g., autotrophs, heterotrophs, diazotrophs, nitrifiers and denitrifiers) would respond to eCO_2_ differentially between soil depths (0–5 cm and 5–15 cm). Our results demonstrated that eCO_2_ had significant effects on the functional structure and metabolic potential of soil microbial communities with similar trends in both soil depths, and that many key functional genes involved in C, N, and P cycling were stimulated by eCO_2_. This study provides new insights into our understanding the response of soil microbial communities to eCO_2_ in this C_4_ agroecosystem.

## Resutls

### Effects of eCO_2_ on plant yield and soil parameters

The grain biomass increased 12.5% when grown at eCO_2_ compared to aCO_2_, although this difference was not statistically significant (*P* = 0.25). The effects of eCO_2_ on some soil properties were different between soil depths. For example, soil NO_3_^−^ level was significantly (*P* < 0.01) decreased in the depth of 0–5 cm under eCO_2_ compared to aCO_2_ but was significantly (*P* < 0.05) increased in the depth of 5–15 cm under eCO_2_. Soil moisture was not significantly different between two CO_2_ treatments in the depth of 0–5 cm, but was significantly (*P* < 0.05) increased in the depth of 5–15 cm at eCO_2_. eCO_2_ did not show any significant impacts on total C, total N, C:N ratio, or NH_4_^+^ contents at either soil depth (Table 1).

### Microbial metabolic potential

**Figure 1:**
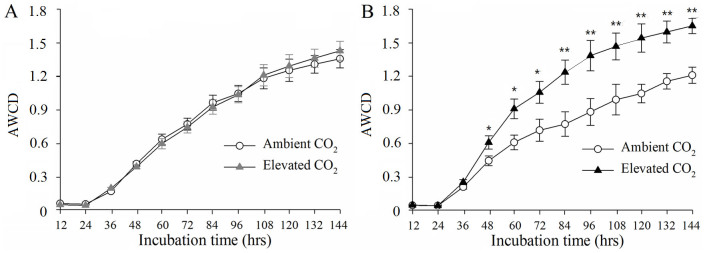
Average well color development (AWCD) of the elevated CO_2_ (eCO_2_) and ambient CO_2 _(aCO_2_) samples in the soil depths of 0–5 cm (A) and 5–15 cm (B) measured by EcoPlate system. Error bars indicate ± SE (standard error) of the four blocks within each depth (*n* = 4). *: *P*< 0.05; **: *P*< 0.01 based on t-test between aCO_2_ and eCO_2_ at each time point.

The metabolic capacity of soil communities collected from eCO_2_ and aCO_2_ conditions were similar across the incubation period of 144 hr in the soil depth of 0–5 cm (Figure 1). However, a significant (*P* < 0.05) stimulation of microbial C utilization capacity by eCO_2_ was observed in the soil depth of 5–15 cm after 48 hr of incubation and such eCO_2_-stimulated effects became greater overtime and lasted until the end of incubation (Figure 1).

### Overview of functional and phylogenetic structure of soil microbial communities

**Figure 2:**
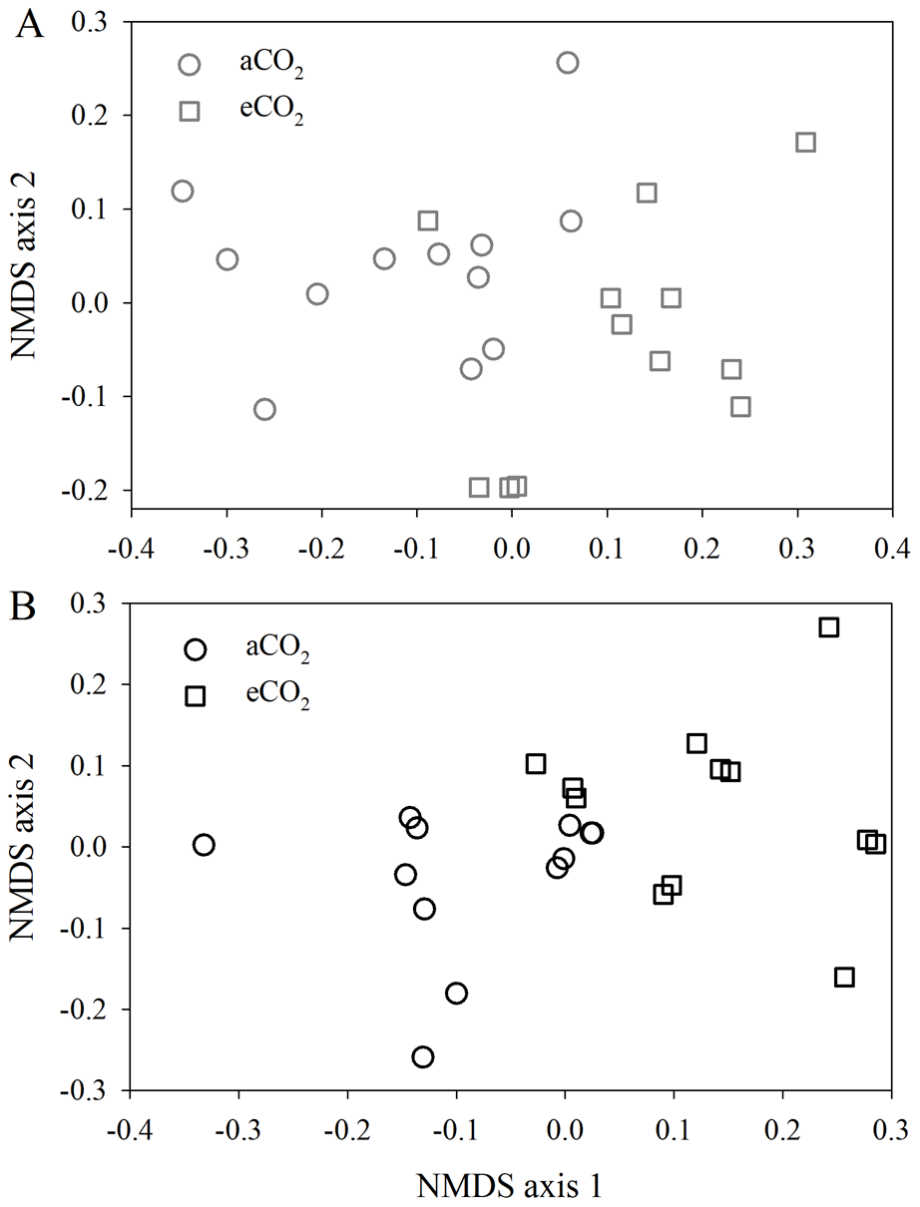
Non-metric multidimensional scaling (NMDS) analysis of elevated CO_2_ (eCO_2_) and ambient (aCO_2_) samples in the soil depths of 0–5 cm (A) and 5–15 cm (B) based on Bray–Curtis values of detected functional genes (*n* = 12).

A total of 6,491 genes were detected across 48 samples. The average number of detected genes (i.e., richness) was significantly (*P* = 0.044) greater (2,816 ± 200) under eCO_2_ than under aCO_2_ (2,202 ± 279) in the soil depth of 0–5 cm. This difference was even greater (*P* < 0.001) in the soil depth of 5–15 cm: 3,463 ± 189 genes detected under eCO_2_, 1,388 ± 137 genes detected under aCO_2_. Non-metric multidimensional scaling (NMDS) analysis based on the Bray-Curtis distance revealed that eCO_2_ dramatically altered the functional structure of microbial communities at both soil depths (Figure 2), and this was also the case for the phylogenetic structure based on the detected *gyrB* genes on GeoChip (Figure S1). Mantel tests indicated the phylogenetic structure was significantly correlated (*r* = 0.813, *P* < 0.001) with the functional structure. Those patterns were also confirmed by dissimilarity tests, showing significantly distinct functional structures between aCO_2_ and eCO_2_ at both depths, or between soil depths at both CO_2_ levels (Table 2). In addition, PERMANOVA revealed that eCO_2_ contributed 11.9% (*P* = 0.001) of the total variation of functional gene structure, while depth explained 5.2% of the variation (*P* = 0.014), and their interaction explained 3.8% (*P* = 0.034) (Table 3). Similarly, we observed significant differences in the phylogenetic structure due to eCO_2_ and depth (Table 3). Furthermore, such a pattern was observed at the functional gene category level, including C, N, P and CH_4_ cycling genes (Table S1).

Collectively, these results revealed that the diversity, composition, structure and functional potential of soil microbial communities were predominantly affected by eCO_2_ in this maize agroecosystem.

### Genes involved in C cycling

A substantial number of Rubisco genes (74 from the soil depth of 0–5 cm and 58 from the depth of 5–15 cm) involved in C fixation were detected, and the abundance (signal intensity) of these genes was significantly (*P* < 0.05) higher under eCO_2_ than under aCO_2_ at both depths (Figure S2). Likewise, under eCO_2_, 12 unique *rbcL* genes were detected in the soil depth of 0–5 cm, while 27 unique genes were detected in the soil depth of 5–15 cm, compared with aCO_2_ at each depth (data not shown). Genes from the other two CO_2_ fixation pathways, CODH and Pcc/Acc, had significantly increased abundances under eCO_2_ in the soil depth of 5–15 cm, but their signal intensities did not differ significantly between two CO_2_ levels in the soil depth of 0–5 cm (Figure S2).

**Figure 3:**
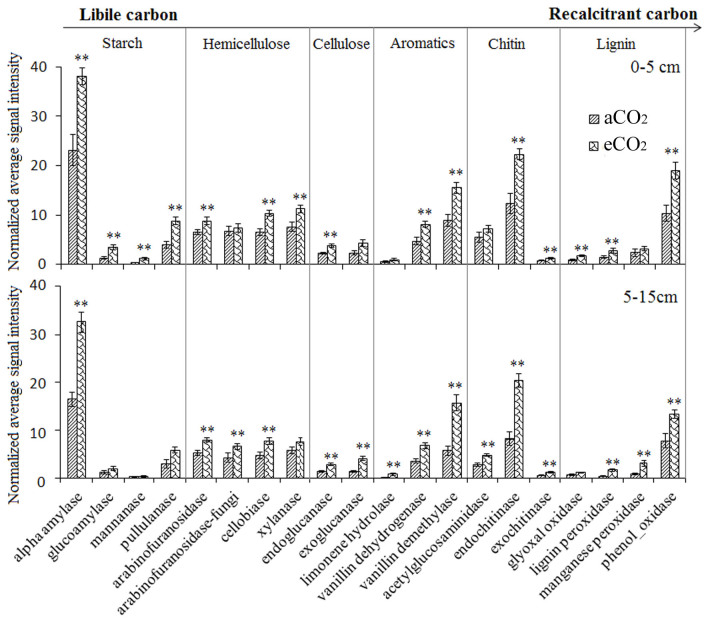
The abundance of detected key genes involved in C degradation. All data are presented as the mean ± SE (standard error, *n* = 12). *: *P* < 0.05; **: *P* < 0.01based on t-test t between aCO_2_ and eCO_2_.

Cellulose, hemicellulose and lignin are the most abundant C sources derived from plant tissues in soil ecosystems. Here, most C degradation genes were significantly (*P*< 0.05) increased under eCO_2_ at both depths (Figure 3). For example, alpha-amylase, cellobiase, endoglucanase, vanillin dehydrogenase, endochitinase and phenoloxidase were all stimulated under eCO_2_. However, some genes responded differently to eCO_2_ along the soil depths (Figure 3). For example, the abundances of all four detected starch degradation genes were significantly (*P* < 0.05) increased under eCO_2_ in the soil depth of 0–5 cm, while only signal intensity of alpha-amylase was increased significantly under eCO_2_ in the soil depth of 5–15 cm. In addition, eCO_2_ increased the abundance of genes involved in CH_4_ cycling, including *mcrA* for methane production, and *pmoA* and *mmoX* genes for methane consumption (Figure S3). Apart from *mmoX*, where the abundance was significantly increased only in the soil depth of 5–15 cm, the significant increases of these genes were observed at both soil depths.

### Genes involved in N cycling

**Figure 4:**
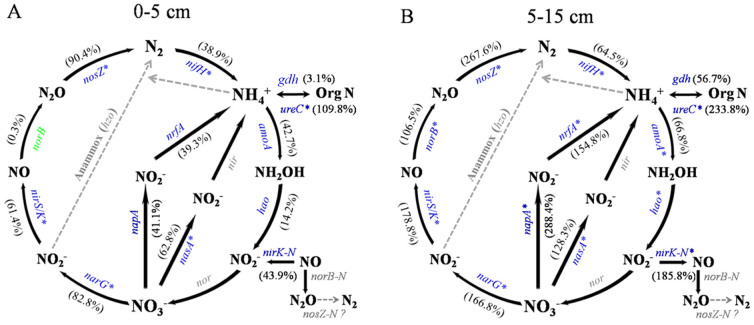
The relative changes of the detected genes involved in N cycling at eCO_2_ compared to aCO_2_. A: soil depth of 0–5 cm; B: soil depth of 5–15 cm soil. The signal intensity for each gene detected was normalized by all detected gene sequences using the mean. The percentage of a functional gene in a bracket was the sum of the signal intensity of all detected sequences of this gene divided by the grand sum of signal intensities of the detected N cycling genes, and weighted by the fold change of the signal intensity of this gene at eCO_2_ to that at aCO_2_. For each functional gene, colors mean that this gene had a higher (blue) or lower (green) signal intensity at eCO_2_ than at aCO_2_ with significance at *P* < 0.05 (*). Gray-colored genes were not targeted by this GeoChip, or not detected in those samples. It remains unknown if *nosZ* homologues exist in nitrifiers. Genes and their involved functional processes: N_2_ fixation by *nifH* encoding nitrogenase; Nitrification by *amoA* encoding ammonia monooxygenase; Denitrification by *narG* encoding nitrate reductase, *nirS* and *nirK* encoding nitrite reductase, *norB* encoding nitric oxide reductase, and *nosZ* encoding nitrous oxide reductase; Dissimilatory N reduction to ammonium by *napA* for nitrate reductase and *nrfA* for c-type cytochrome nitrite reductase; Ammonification by *gdh* encoding glutamate dehydrogenase and *ure C* encoding urease; Assimilatory N reduction, *nasA* encoding nitrate reductase.

A total of 519 and 574 genes involved in N cycling were detected under aCO_2_ and eCO_2_, respectively, in the soil depth of 0–5 cm, and 287 and 570, respectively in the soil depth of 5–15 cm. eCO_2_ significantly (*P* < 0.05) increased the abundance of genes involved in N fixation (*nifH*), ammonification (*ureC*), denitrification (*narG*, *nirS/K* and *nosZ*) and assimilatory N reduction (*nasA*) at both depths (Figure 4A and 4B). Additionally, signal intensities of genes involved in nitrification (*amoA* and *hao*), and dissimilatory N reduction to ammonium (*napA* and *nrfA*) were only enhanced under eCO_2_ in the soil depth of 5–15 cm (Figure 4B).

### Genes involved in P cycling

GeoChip 3.0 targets genes involved in exopolyphosphatase (Ppx) for inorganic polyphosphate degradation, polyphosphate kinase (Ppk) for polyphosphate biosynthesis in prokaryotes, and phytase for phytate degradation. Abundances of Ppk and Ppx genes were significantly increased (*P* < 0.05) under eCO_2_ compared to aCO_2_ at both depths, while phytase genes were significantly increased in the soil depth of 0–5 cm under eCO_2_ but remained unchanged in the soil depth of 5–15 cm (Figure S4).

### Linking microbial functional structure to soil properties

Mantel tests were performed to examine the correlation between the microbial community structure and soil properties (TC, TN, NO_3_^−^, NH_4_^+^, and C:N ratio) and corn yield, no significant correlations were detected when all detected genes were considered. We then calculated the correlation between functional categories and soil variables. The results revealed that TN and TC were significantly (*P* < 0.05) correlated with the microbial community structure based on N fixation and nitrification genes, respectively (Table S2), whereas NO_3_^−^, NH_4_^+^and C/N ratio did not show significant correlations at the functional category level. We further examined the correlation of soil properties with individual functional gene families, and found that 30 functional gene families had significant (*P* < 0.05) correlations with soil properties, including those involved in C degradation, N cycling, CH_4_ consumption, bioremediation of aromatics, herbicides and pesticides (Table S3). For example, genes involved in C degradation (acetylglucosaminidase, pectinase, xylanase and *amyA*genes), denitrification (*norB*), methane consumption (*mmoX*) and bioremediation/biodegradation of aromatics (*pheA*, *oxdB*, *alkB* and *nagL*), herbicides (*pcpA* and *mhpC*) and hydrocarbons (*cpnA*) were significantly (*P* < 0.05) correlated with soil properties, such as TC, TN and C:N ratio, and corn yield (Table S3).

## Discussion

As soil microorganisms play important roles in mediating ecological processes (e.g., nutrient cycling, plant growth), understanding the response of soil microbial communities to eCO_2_ is critical to fully assess the impact of eCO_2_ on ecosystem functioning and stability and predict future climate change. In this study, we demonstrated that the functional structure and metabolic potential of microbial communities in a C_4_ maize agroecosystem were significantly altered under crops fumigated with eCO_2_ for eight years. The significant response to eCO_2_ was observed in samples of both soil depths, although some different microbial responsive patterns were observed between the two soil depths, and the abundance of many key functional genes was significantly increased and correlated with soil properties (e.g., nitrate, ammonia, soil C and N). CO_2_ was the dominant factor shaping the soil microbial functional structure, although depth and their interaction had significant contributions as well. Therefore, this study provides new insights into our understanding the response of soil microbial communities to eCO_2_ in C_4_ plant agroecosystems.

**Figure 5:**
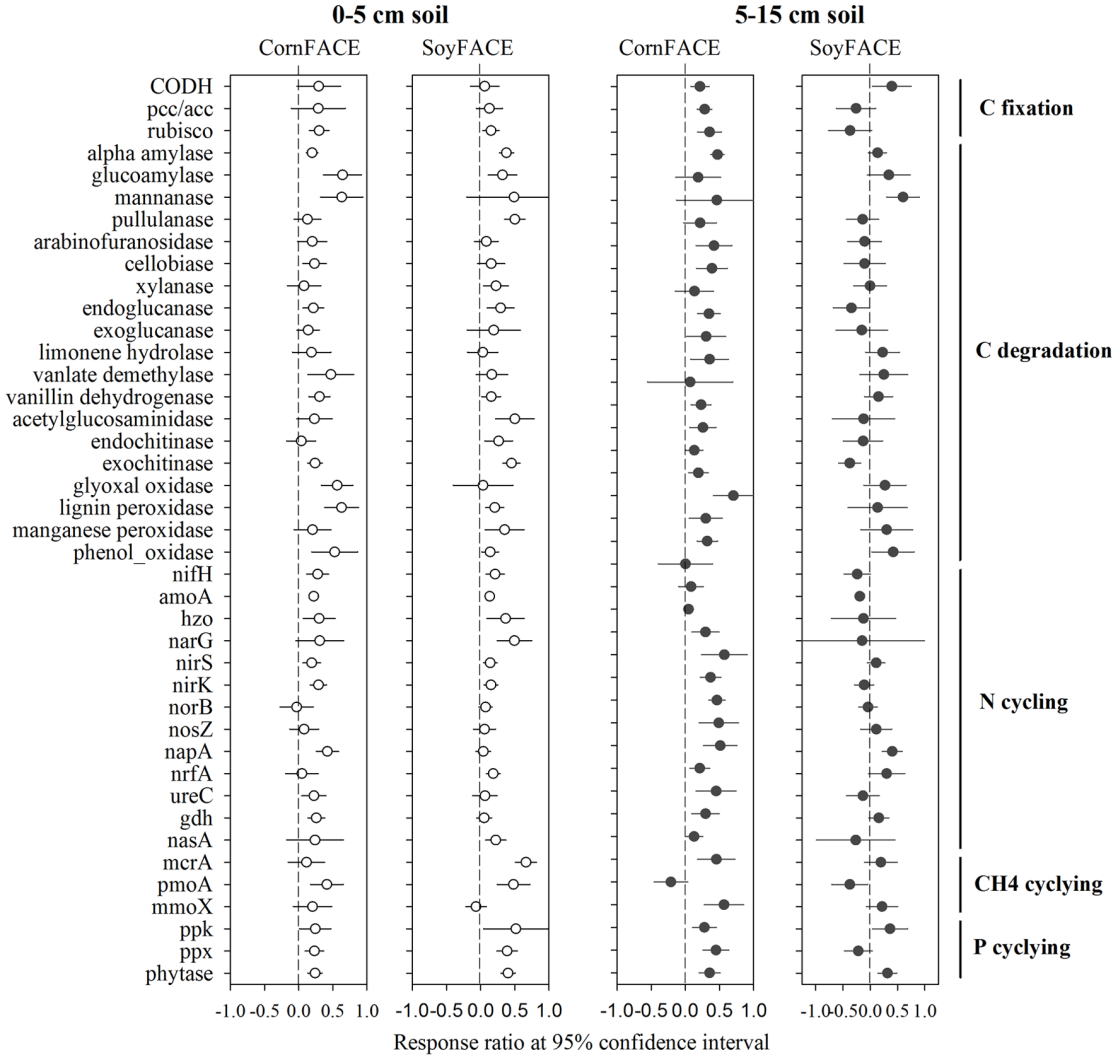
Comparisons of effects of eCO_2_ on the abundance of functional genes with maize and soybean crops in the soil depths of 0–5 cm (open circle) and 5–15 cm (solid circle). Significance was determined using the response ratio analysis^5^ at a 95% confidence interval (CI).

The C_4_ maize system examined in this study, and the C_3_ soybean plots that were the subject of a previous study^7^ were established in 2001 at the SoyFACE site (Champaign, IL, USA), and operated under typical Midwestern crop management practices^21^,^38^. While both studies revealed similar responses of soil microbial communities to eCO_2_ in the depth of 0–5 cm, a much greater stimulation of abundances of key functional genes involved in C and N cycling was observed in the depth of 5–15 cm in the maize field compared to the soybean field (Figure 5). This discrepancy may be due to the eCO_2_ enhanced water use efficiency for maize plants that would mitigate the drought stress on microbial activities^21^. Taken together, our data indicated that eCO_2_ may have comparable or even greater effects on microbial functional potential associated with C_4_ crops in comparison to C_3_ crops, which has not been recognized so far, which highlights the significance of this study.

Elevated CO_2_ largely impacts soil microbial communities indirectly through increased plant C input, altered plant litter quality (e.g., C%, N%), and/or modified soil properties (e.g., soil pH and moisture)^2^,^4^,^15^,^22^. In the limited number of studies on C_4_ plants under eCO_2_, the stimulation of plant biomass and/or alteration of litter quality under eCO_2_ have not been previous observed^21^,^23^,^24^,^25^. Similarly, we did not detect a significant change in maize yield under eCO_2_ in this study. However, eCO_2_ could significantly improve, although the effect was small, C_4_ plant water use efficiency by reducing midday stomatal conductance or under drought^26^,^27^, thus indirectly stimulating the growth of C_4_ plants by delaying and ameliorating drought stress^10^. In 2008, the SoyFACE site experienced ‘atypical' precipitation year with wet early-season, a single extended drying event in the mid-season, and rewet late-season^25^. Soil samples used in this study were collected in the drought period in August 2008. The drought event could cause physiological stresses for maize plants, but growth under eCO_2_ might delay or relieve drought-induced reduction of net photosynthetic CO_2_ uptake, resulting in higher leaf-level photosynthetic C gain in the drought season^25^. The enhanced photosynthetic C gain, eventually increased plant C input may enhance C allocation into belowground in the form of root biomass and rhizodeposits under eCO_2_, which may result in the alteration of microbial functional structure and metabolic potential, and especially the stimulation of C degradation and N cycling genes.

Given the previously reported conservation of soil moisture by C_4_ crops under eCO_2_^21^, we hypothesize that microbial activities were constrained by drought conditions under C_3_ plants, while the water-use efficiency of C_4_ plants mitigated the drought effects on microbial activities under the maize cropping system^21^,^26^. A previous study has demonstrated that the efflux of amino acids from maize roots was significantly enhanced under eCO_2_, although plant biomass remained unchanged^28^. Also, greater soil moisture under eCO_2_, due to the improved water use efficiency of C_4_ plants under elevated CO_2_^25^, may alter the microbial community structure and function, particularly under drought condition^29^,^30^,^31^. For example, it has been shown that soil microbial activity was consistently enhanced in tallgrass prairie under eCO_2_ treatment due to improved soil water condition, which was closely correlated with soil water content in the depth of 0–5 cm^19^. Indeed, in this study, we found that soil moisture was significantly greater under eCO_2_ in the depth of 5–15 cm. Therefore, although it is beyond our research scope to identify mechanisms explaining how eCO_2_ shifts the microbial community structure and function, this study supports our hypothesis that eCO_2_ would alter the functional composition, structure, and metabolic potential of soil microbial communities associated with maize cultivation, possibly through increased soil moisture. Indeed, a recent study also showed that eCO_2_ increased soil moisture along with decreased maize evapotranspiration by 7–11%^32^. We expect this effect to be more evident under drought conditions^21^. Therefore, in this study, our results support the above hypothesis, evidenced by the comparison of the effects of eCO_2_ on the abundance of functional genes between maize and soybean crops. That is, eCO_2_ substantially stimulated the functional gene abundances at both depths in this maize FACE experiment (Figure 5), but only minor eCO_2_ effects were detected in the 5–15 cm soil planted with soybean at the SoyFACE site^7^.

It is also hypothesized that various microbial functional groups (e.g., C fixers, C degraders, diazotrophs and denitrifiers) would be stimulated differentially between two soil depths by eCO_2_. This study indicated that the abundance of key functional genes involved in C, N and P cycling was significantly stimulated under eCO_2_ at both soil depths. First, eCO_2_ increased the signal intensity of 75% (15 out of 20) of the detected functional genes involved in C degradation at both soil depths (0–5 cm and 5–15 cm), with 11 genes responding positively to eCO_2_ in two depths. These ‘common' eCO_2_-enriched genes in both soil depths, which are capable of decomposing a variety of C compounds present in plant materials and soil organic matter. Such responses of soil microbial communities to maize fumigated with eCO_2_ are generally consistent with previous studies^2^,^6^,^7^. The significantly enhanced C degradation genes under eCO_2_ may indicate the stimulation of microbially-mediated C decomposition in soil. However, this does not imply that soil C storage was reduced under eCO_2_, since soil C storage and stability are also largely affected by other factors, such as plant C input (e.g., quality and quantity), plant nutrient uptake (e.g., N, P), soil properties, and size and turnover of different C pools (e.g., native soil C pool, fresh plant litter pool, microbial C pool)^2^,^5^,^23^,^33^,^34^. Although some studies showed that eCO_2_ led to the loss of soil C^8^,^9^, other studies showed an increase in soil C^35^, or no significant effects on soil C content^2^,^36^, which is consistent with the current study. Especially in this SoyFACE, previous studies showed that management practices affected soil C and N stocks and dynamics more than eCO_2_-stimulated effects^34^,^37^. Such common responses at both soil depths are also reflected in N cycling genes. Although N fertilizer was yearly applied to the maize plots before planting^38^, key genes involved in N fixation (*nifH*) and ammonification (*ureC*) were significantly increased under eCO_2_ compared to aCO_2_. This finding agrees with other studies showing that microbial N fixation or the abundance of N fixation genes increased under eCO_2_^2^,^5^,^39^. If such increased gene abundances are translated to increased N fixation and ammonification process rates, this may relieve progressive N limitation observed previously in other FACE sites^40^,^41^,^42^,^43^. Also, key genes involved in denitrification were generally stimulated under eCO_2_. For example, a previous study showed that the *nirK* abundance increased more than doubled while its diversity was significantly reduced in soil where trembling aspen was grown undereCO_2_^44^. In contrast, the signal intensity of denitrification genes was not generally stimulated under eCO_2_ in a soybean agroecosystem (Figure 5)^7^, and a similar pattern was detected by qPCR analysis of *amoA* and *nosZ* gene abundances at this site in the same year, showing that eCO_2_ has limited effects on N transformations in soybean agroecosystem^45^. Based on the measurement of N_2_O fluxes, several studies have reported that denitrification is enhanced at eCO_2_^46^,^47^, although exceptions were also reported^48^. Such a difference may be complicated by other factors, such as soil moisture, type and aggregate size. For example, the abundance of *nosZ* genes increased in the microaggregates under reduced precipitation but not by eCO_2_ or in the whole soil compared to ambient conditions^34^. If the increased denitrification genes indicate an enhanced denitrification processes under eCO_2_, this may result in increased N_2_O consumption and production, a possible positive feedback to global change.

However, some differential responses of soil microbial communities to eCO_2_ were also detected between the two soil depths. First, three key C fixation genes from three pathways, including Rubisco for the Calvin cycle, CODH for the reductive acetyl-CoA pathway, and PCC/ACC for the 3-hydroxypropinate/malyl-CoA cycle, increased significantly under eCO_2_ in the soil depth of 5–15 cm, while only Rubisco genes increased significantly in the depth of 0–5 cm. Second, the abundance of genes involved in nitrification (*amoA* and *hao*) and dissimilatory N reduction (*napA* and *nrfA*) was significantly enhanced in the soil depth of 5–15 cm, but unchanged in the depth of 0–5 cm. Third, the microbial metabolic potential as measured by EcoPlate increased significantly under eCO_2_ in the soil depth of 5–15 cm, but was similar in the depth of 0–5 cm. Also, the abundances of four starch degradation genes detected by GeoChip were all significantly increased under eCO_2_ in the soil depth of 0–5 cm, but only alpha-amylase gene was stimulated in the depth of 5–15 cm. Additionally, the response of P cycling genes to eCO_2_ appeared greater in the soil depth of 0–5 cm than that of 5–15 cm.

Several reasons may contribute to the subtle difference in microbial responses to eCO_2_. First, the composition and functional structure of microbial communities were significantly different between the two soil depths, thus resulting in differential functional potential/activity. Second, soil may contain more organic matter from plant residues in the depth of 0–5 cm than that of 5–15 cm, resulting in differences of nutrient availability for microbial growth and activities. Third, many soil physiochemical properties (e.g., O_2_ concentration, soil aggregate size, pH, moisture, temperature) may change with depths, and some of them (e.g., soil moisture, temperature) may experience wider fluctuations in the depth of 0–5 cm soil than that of 5–15 cm^16^,^17^,^18^, thus differentially affecting microbial responses to eCO_2_^19^,^49^,^50^. For example, it has been shown that soil microbes responded differently to eCO_2_, warming, and their interactions^49^. Similarly, Castro and colleagues studied how microbes responded to multiple climate change factors (eCO_2_, warming and precipitation) and found complex responsive patterns with multiple factors^50^. Therefore, our results showed that microbial responses to eCO_2_ were consistent overall, and soil depth only had a minor effect, indicating eCO_2_ had a much greater impact on microbial structure and function than soil depth.

In summary, this study highlights the necessity and importance of examining the microbial response to eCO_2_ in C_4_ agroecosystem. The significant stimulation of a great number of key functional genes involved in C and N cycling at both soil depths may indicate the potential of altered C and N dynamics in soils planted with C_4_ crops. This study provides new insights into our understanding of soil microbial community responses to eCO_2_ in a C_4_ maize agroecosystem. However, further studies are needed to understand the mechanism by which microbial structure and function shift at the eCO_2_ environment and their feedbacks to ecosystem functioning, stability and services, especially with different C_4_ plant species.

## Methods

### Site description and sample collection

The SoyFACE experimental site at Champaign, IL, USA (40°03′N, 88°12′W, 228 m above sea level) was established in 2001 on tile-drained farmland that had been under cultivation for over 100 years. The crops are rotated between maize (*Z. mays* cv. 34B43, Pioneer Hi-Bred International) and soybean (*Glycine max*) on a yearly basis. The soil is Drummer–Flanagan series (fine-silty, mixed, mesic Typic Endoaquoll) and organic rich^38^. Fertilizer was applied to maize fields yearly at a rate of 202 kg N ha^−1^ (157 kg N ha^−1^ with 28% 1:1 urea: ammonium nitrate liquid before planting and 45 kg N ha^−1^ credit from previous soybean N_2_ fixation)^38^. Atmospheric CO_2_ of four replicate plots (each with a 20-m diameter) was maintained at the ambient CO_2_ (aCO_2_, 354 ppm) level, and four replicates are maintained at an elevated (~550 ppm) CO_2_ level in a randomized complete block design. To minimize the cross-contamination, aCO_2_ and eCO_2_ plots in each block were set with a 100-m interval. Three subsamples were collected in each plot at two soil depths (0–5 cm and 5–15 cm) from both CO_2_ treatments before harvest in August 2008, resulting in a total of 48 samples (4 plots x 3 subsamples x 2 CO_2_ treatments x 2 depths). Soil samples were sieved through a 2-mm sieve to remove visible plant materials. All soil samples were immediately stored at 4°C or −80°C until soil property analysis or DNA extraction.

### Plant and soil analysis

Plant yields (grain biomass) were collected and analyzed at the end of the growing season. Soil total C and total N were measured by combustion (Muti N/C 3100, Jena, Germany). Soil NO_3_^−^ and NH_4_^+^ were extracted with 20 ml of 2 M KCl and analyzed using a segmented flow analyzer (Skalar Sanplus, Breda, Netherlands).

### Analysis of microbial metabolic potential

BioLog EcoPlate^TM^ substrate utilization assays^51^ containing 31 sole carbon sources and control wells (without substrates) with three replicates in a 96-well plate were used to evaluate the metabolic potential of soil microbial communities. Three subsamples collected from the same plot were composited together, resulting in 4 biological replicates from aCO_2_ and eCO_2_ at both depths. The soil suspension was prepared by adding 5.0 g soil to 45 ml of double distilled H_2_O, followed by shaking for 45 min with 200 rpm at 4°C. Then samples were allowed to settle for 30 min before the supernatant was collected and serially diluted to 10^−4^ based on a pilot experiment. An aliquot of 100 µl of the diluted suspension from each soil sample was then inoculated into each EcoPlate well, and incubated at 25°C for 168 hours with in an OminLog System (BioLog Inc., Hayward, CA, USA). Well color development was automatically measured by OminLog System at 15 min intervals during the incubation. The average well color development (AWCD) presents the potential utilization of various carbon sources by a microbial community. AWCD was calculated by the differences between the OD_590_ of the wells containing individual carbon sources and the control wells according to AWCD = ∑(C-R)/31, where C is the OD_590_ value of each well, R is the OD_590_ value of the control well^52^.

### DNA extraction

DNA was extracted from 5.0 g of soil samples using the method described previously^53^. DNA quality was assessed by the ratio of 260/280 nm and 260/230 nm using a ND-1000 spectrophotometer (NanoDrop Inc., Wilmington, NC) and DNA concentration was quantified with a Quant-It^TM^ PicoGreen (Invitrogen, Carlsbad, CA).

### GeoChip analysis

Purified DNA was amplified using whole community genome amplification (WCGA) and labeled with fluorescent dyes as described previously^54^,^55^. The labeled DNA was then hybridized to GeoChip 3.0 at 42°C for 12 hrs^56^. After hybridization, the chips were scanned using a ScanArray 5000® Microarray Analysis System (PerkinElmer, Wellesley, MA) at 95% laser power and 75% PMT (photomultiplier tube gain), and the signal intensity of each spot was measured using ImaGene™ 6.1 Standard Edition (Biodiscovery Inc., El Segundo, CA). Spots with signal-to-noise ratio (SNR) < 2.0 were removed. Probe signal intensities were normalized by their own universal standards in the experiment. A probe was considered positive if it was detected in at least 3 out of 12 replicates. These positive spots were included for further analysis.

### Statistical analysis

An unpaired t-test was conducted to test the significances of plant yield between aCO_2_ and eCO_2_, soil variables between aCO_2_ and eCO_2_ in each soil depth (0–5 cm or 5–15 cm), microbial metabolic potential between aCO_2_ and eCO_2_ at each time point in each soil depth, respectively^57^. The functional structure of microbial communities was ordinated using the NMDS based on the Bray-Curtis distance^58^. Non-parametric permutational multivariate analysis of variance (PERMANOVA), analysis of similarities (ANOSIM), and multi-response permutation procedure (MRPP) were used to evaluate the significance of the functional structure between aCO_2_ and eCO_2_ at each depth based on the null hypothesis^58^,^59^. The effect of eCO_2_ on the abundance of a given functional gene was analyzed by computing the response ratio^5^. Also, non-parametric permutational multivariate analysis of variance (PERMANOVA) was conducted to quantitatively evaluate the contribution of CO_2_ and depth to the microbial community structure using the ‘adonis' function^58^. Mantel tests were used to examine the correlation between the microbial community structure and environmental factors (soil properties and corn yield). All of the above analyses were performed with R v.2.8.1 project in the vegan package (v.1.15-1) (www.R-project.org).

## Author Contributions

All authors contributed to the data set, discussed the results and commented on the manuscript. Z.H., A.K. and J.Z. designed this study. J.X. did the experiments, and J.X. and Y.D. did data analysis. J.X., Z.H. and S.S. wrote this paper with help from A.K. J.D.V., L.W. and J.Z.

## Supplementary Material

Supplementary Information Supplemental Information

## Figures and Tables

**Table 1:** Effects of eCO_2_ on soil properties at both depths

		Moisture	NO_3_^−^-N	NH_4_^+^-N	Total nitrogen	Total carbon	
		(%, w/w)	(mg/kg)	(mg/kg)	(w/w, %)	(%, w/w)	TC/TN ratio
0–5 cm	aCO_2_	24.0 ± 2.5^B^	1.28 ± 0.11^A^	30.4 ± 2.82^A^	0.164 ± 0.011^A^	2.43 ± 0.192^A^	15.23 ± 0.90^A^
	eCO_2_	24.1 ± 2.7^b^	0.89 ± 0.06^b^	36.4 ± 4.07^a^	0.165 ± 0.013^a^	2.12 ± 0.148^a^	13.85 ± 0.61^a^
	*P*	0.712	**0.030**	0.418	0.328	**0.042**	0.813
5–15 cm	aCO_2_	36.5 ± 2.7^A^	1.04 ± 0.07^B^	32.61 ± 1.90^A^	0.155 ± 0.008^A^	2.24 ± 0.258^A^	13.37 ± 0.79^A^
	eCO_2_	38.5 ± 2.4^a^	2.52 ± 0.59^a^	31.34 ± 2.50^a^	0.148 ± 0.007^a^	1.91 ± 0.101^a^	13.04 ± 0.51^a^
	*P*	**0.037**	**0.023**	0.267	0.879	0.823	0.615

Soil variables from each depth were analyzed separately and significances between treatments (aCO_2_ and eCO_2_) or two soil depths were tested by *t*-test at the *P*< 0.05 level. A and B indicate significant changes between depths for aCO_2_, and a and b for eCO_2_.

**Table 2:** Significance tests of the effects of CO_2_ and depths on the overall microbial community structure with three different statistical approaches

		aCO_2_ vs. eCO_2_		0–5 cm vs. 5–15 cm	
		0–5 cm	5–15 cm	aCO_2_	eCO_2_
Adonis^a^	*F*	0.108	0.228	0.118	0.085
	*P*	**0.008**	**0.001**	**0.007**	**0.032**
ANOSIM^b^	*R*	0.210	0.424	0.115	0.055
	*P*	**0.004**	**0.001**	**0.014**	0.134
MRPP^c^	*δ*	0.514	0.453	0.483	0.484
	*P*	**0.005**	**< 0.001**	**0.006**	**0.022**

^a^Non-parametric permutational multivariate analysis of variance (PERMANOVA) with the adonis function;

^b^Analysis of similarities ANOSIM;

^c^Non-parametric procedure that does not depend on assumptions such as normally distributed data or homogeneous variances, but rather depends on the internal variability of the data.

**Table 3:** The effects of eCO_2_ and soil depth on the functional and phylogenetic structure of soil microbial community by non-parametric permutational multivariate analysis of variance (PERMANOVA) with the *adonis* function. The functional structure data were based on all detected genes by GeoChip while the phylogenetic structure data were based on *gyrB* only. R^2^ value is the constrained percentage of the parameter

	CO_2_		Depth		CO_2_:Depth	
	R^2^	*P*	R^2^	*P*	R^2^	*P*
Functional structure	0.119	**0.001**	0.052	**0.014**	0.038	**0.034**
Phylogenetic structure	0.103	**0.001**	0.049	**0.016**	0.027	0.155
