# Supplementary material for: Elevated CO2 shifts the functional structure and metabolic potentials of soil microbial communities in a C4 agroecosystem
Source: Sci Rep. 2015 Mar 20;5:9316. doi: 10.1038/srep09316 (PMC4366761; doi:10.1038/srep09316)
Supplement: Supplementary Information — Supplemental Information [file srep09316-s1.pdf]

Supplemental Information

Elevated CO_2 shifts the functional structure and metabolic potentials of soil microbial communities in a C_4 agroecosystem

JinboXiong, Zhili He, Shengjing Shi, Angela Kent, Ye Deng, Liyou Wu, Joy D. Van Nostrand, and Jizhong Zhou

A. Supplemental Tables

Table S1 Quantitatively evaluates the effects of eCO_2 and soil depths on the functional gene categories by parametric permutational multivariate analysis of variance (perMANOVA)

Table S2 The correlation of functional process involved in soil C and N dynamics with soil variables by Mantel test

Table S3 P values of correlations between soil properties and signal intensities of functional genes by Mantel tests

B. Supplemental Figures

Figure S1 Non-metric multidimensional scaling (NMDS) analysis of elevated CO_2 (eCO_2) and ambient CO_2 (aCO_2) samples

Figure S2 The normalized signal intensity of the detected key gene families involved in CO_2 fixation

Figure S3 The normalized signal intensity of the detected key gene families involved in CH_4 cycling

FigureS4 The abundance of detected key genes involved in P utilization

Table S1 Quantitativelyevaluates the effects of eCO_2 and soil depths on the functional gene categories by parametric permutational multivariate analysis of variance (perMANOVA). R^2 value is the constrained proportion of the parameter.

Gene category	eCO_2		Depth		eCO_2:Depth	
	R^2	*P*	R^2	*P*	R^2	*P*
C fixation	0.105	**0.001**	0.056	**0.008**	0.036	0.055
C degradation	0.116	**0.001**	0.054	**0.002**	0.031	0.068
N fixation	0.102	**0.001**	0.048	**0.011**	0.033	0.067
Nitrification	0.056	**0.009**	0.067	**0.007**	0.061	**0.005**
Denitrification	0.129	**0.001**	0.054	**0.009**	0.037	**0.039**
Assimilatory N reduction	0.104	**0.001**	0.046	**0.018**	0.063	**0.002**
Dissimilatory N reduction	0.131	**0.001**	0.052	**0.016**	0.037	0.059
Ammonification	0.093	**0.001**	0.056	**0.001**	0.033	**0.043**
P utilization	0.128	**0.001**	0.048	**0.017**	0.04	**0.023**
CH_4 production	0.127	**0.001**	0.085	**0.001**	0.061	**0.003**
CH_4 oxidation	0.094	**0.001**	0.048	**0.007**	0.031	0.097

Table S2 The correlation of functional process involved in soil C and N dynamics with soil variables by Mantel test.

**Functional category**	TN§		TC	
	r	*p*	*r*	*p*
C fixation	0.074	0.137	-0.017	0.481
C degradation	0.08	0.133	0.032	0.266
N fixation	0.146	**0.024**	0.141	**0.051**
Nitrification	0.183	**0.012**	0.201	**0.031**
Denitrification	0.057	0.192	-0.016	0.487
Ammonification	0.076	0.175	-0.03	0.534
Assimilatory N reduction	0.046	0.235	0.016	0.343
Dissimilatory N reduction	0.067	0.146	-0.043	0.687
CH_4 production	0.105	0.111	-0.116	0.894
CH_4 oxidation	0.166	**0.049**	0.037	0.294
P utilization	0.074	0.113	0.028	0.304
S cycling	0.067	0.151	0.016	0.336

§:The other soil variables, including NO_3^-, NH_4^+, C:N ratio and corn yield that showed no significant trends to any functional processes are not represented in this table.

Table S3 *P* values of correlations between soil properties and signal intensities of functional genes by Mantel test. Bold face indicates significantly changed *P* values ($P < 0.05$).

Gene/enzyme	Function	NO_3^-	NH_4^+	TN	TC	C/N
**0-5 cm soil**						
AceA	C degradation	0.763	0.977	0.179	**0.009**	**0.025**
amyA	C degradation	0.551	0.694	**0.038**	0.1	0.22
norB	Denitrification	0.854	0.965	0.607	0.129	**0.032**
alkB	Aromatics	0.414	**0.006**	0.496	0.646	0.399
alkK	Aromatics	0.288	0.98	0.244	**0.02**	**0.027**
CDD	Aromatics	0.765	0.469	0.391	0.446	**0.048**
nagL	Aromatics	0.265	0.753	0.471	0.072	**0.006**
nhh	Aromatics	0.448	0.909	0.062	**0.009**	0.069
oxdB	Aromatics	0.552	0.926	0.279	0.039	**0.032**
phdG	Aromatics	0.634	**0.008**	0.268	0.463	0.572
pheA	Aromatics	0.922	0.824	**0.032**	**0.02**	0.074
tmoABE	Aromatics	0.211	0.83	0.067	**0.033**	0.099
xylXY	Aromatics	0.211	0.902	0.058	**0.022**	0.061
nphA	Aromatics	0.438	0.365	**0.035**	0.189	0.694
dxnA	Herbicides	0.345	0.364	**0.027**	0.497	0.662
mhpC	Herbicides	0.888	0.947	0.13	**0.031**	0.103
pcpA	Herbicides	0.7	0.694	0.056	**0.017**	**0.046**
pcpE	Herbicides	0.703	**0.049**	0.235	0.389	0.792
cpnA	Hydrocarbons	0.384	0.752	0.126	0.051	0.097
nitA	Hydrocarbons	0.639	0.854	0.165	**0.043**	0.078
bco	Pesticides	0.214	0.393	0.425	0.067	**0.019**
dbdC	Aromatics	0.882	0.618	**0.009**	0.186	0.408
nphA	Aromatics	0.438	0.365	**0.035**	0.189	0.694
**5-15 cm soil**						
Acetylglucosaminidase	C degradation	0.521	0.331	0.367	0.07	**0.037**
ara	C degradation	0.324	0.817	**0.016**	0.251	0.7
Pectinase	C degradation	**0.02**	0.625	0.443	0.062	**0.041**
Xylanase	C degradation	0.973	0.939	**0.004**	0.104	0.534
mmoX	CH4 consume	0.632	0.595	0.142	**0.048**	0.125
dbdC	Aromatics	0.882	0.618	**0.009**	0.186	0.408
nphA	Aromatics	0.438	0.365	**0.035**	0.189	0.694
BADH	Hydrocarbons	0.877	0.727	**0.024**	0.512	0.9
cpnA	Hydrocarbons	0.969	0.703	**0.043**	0.282	0.699
rd	Herbicides	0.18	0.587	**0.045**	0.381	0.666

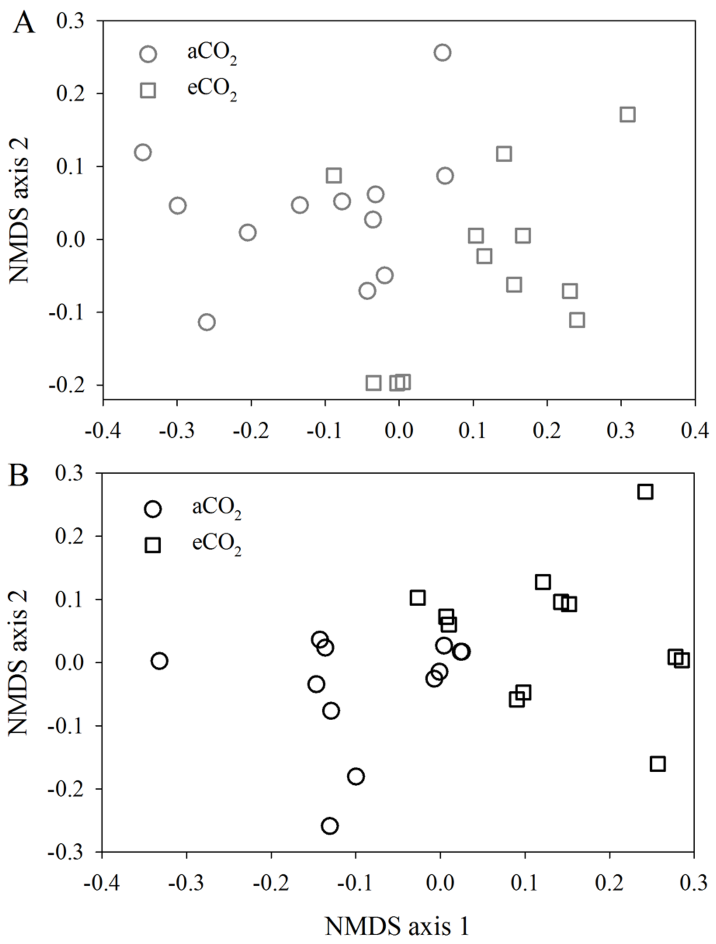

Figure S1 Non-metric multidimensional scaling (NMDS) analysis of elevated CO_2 (eCO_2) and ambient CO_2 (aCO_2) samples at the depths of 0-5 cm (A) and the 5-15 cm (B) based on the Bray–Curtis values of GeoChip detected *gyrB* gene.

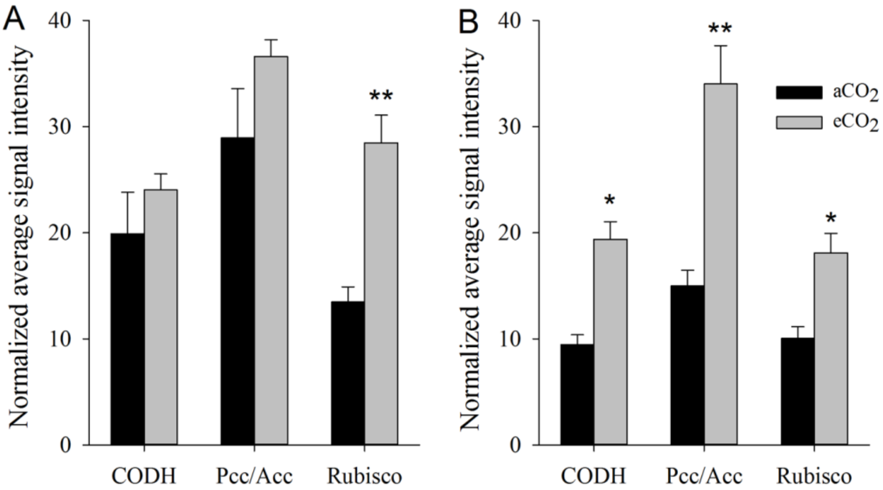

Figure S2 The normalized signal intensity of the detected key gene families involved in CO_2 fixation. Rubisco: ribulosel, 5-bisphosphate carboxylase/oxygenase; CODH: carbon monoxide dehydrogenase; Pcc/Acc: propionyl-CoA/acetyl-CoA carboxylase. Data are presented as mean ± SE (standard error, n = 12). *: $P < 0.05$; **: $P < 0.01$ based on t-test between elevated CO_2 (eCO_2) and ambient CO_2 (aCO_2) samples in the soil depths of 0-5 cm (A) and 5-15 cm (B).

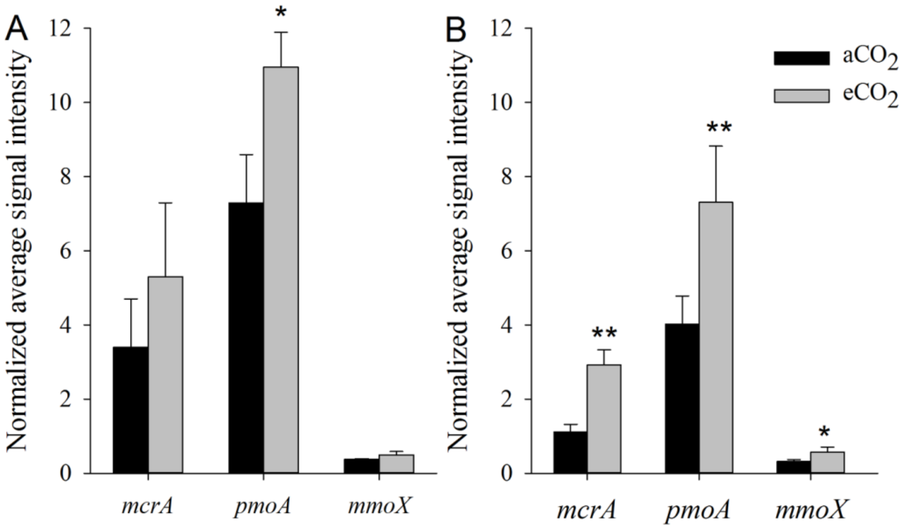

Figure S3 The normalized signal intensity of the detected key gene families involved in CH_4 cycling. *mcrA*: the alpha-subunit of methyl coenzyme M reductase for methane production; *pmoA*: particulate methane monooxygenase, and *mmoX*: methane monooxygenase for methane consumption. Data are presented as mean ± SE (standard error, n = 12). *: $P < 0.05$; **: $P < 0.01$ based on t-test between elevated CO_2 (eCO_2) and ambient CO_2 (aCO_2) samples in the soil depth of 0-5 cm (A) and 5-15 cm (B).

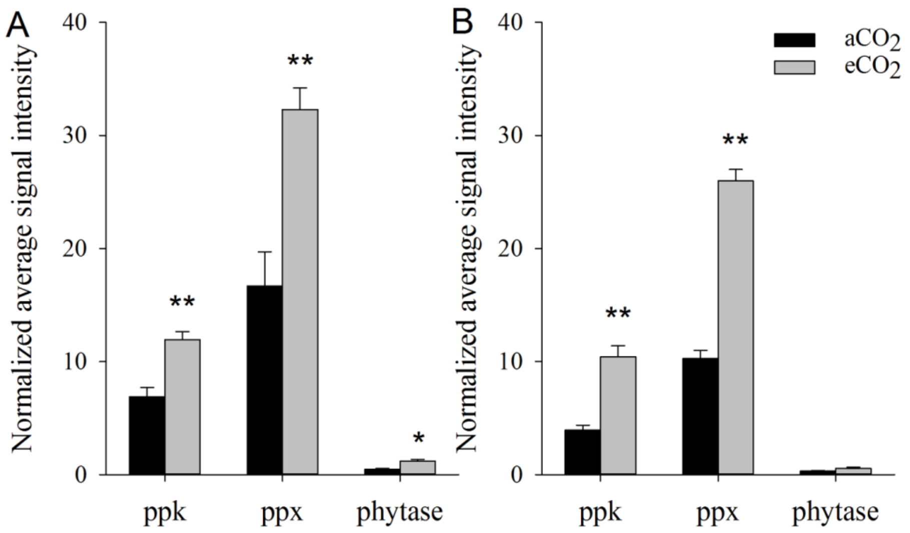

Figure S4 The abundance of detected key genes involved in P utilization in the soil depth of 0-5 cm (A) and 5-15 cm (B). Ppx, exopolyphosphatase for inorganic polyphosphate degradation; Ppk, polyphosphate kinase for polyphosphate biosynthesis in prokaryotes; and phytase for phytate degradation. Data are presented as mean ± SE. *: $P < 0.05$; **: $P < 0.01$ based on the Student t-test.
